# Letter from the Editor in Chief

**DOI:** 10.19102/icrm.2020.111001

**Published:** 2020-10-15

**Authors:** Moussa Mansour


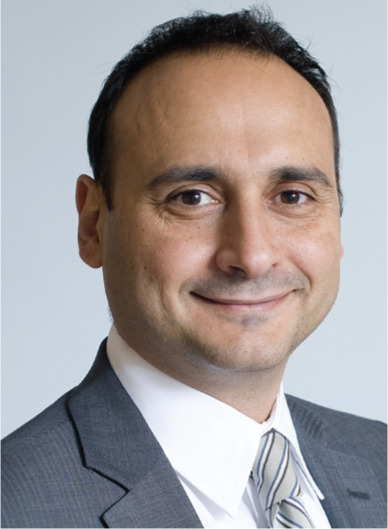


Dear Reader,

This issue of *The Journal of Innovation in Cardiac Rhythm Management* contains many important articles. I would like to highlight the one by Yang et al. titled “Initial Experience with High-density Mapping of Ischemic Ventricular Tachycardia Using a Narrow 0.1-mV to 0.25-mV Border-zone Window.” In it, the authors describe their experience with high-density multielectrode catheter mapping of ventricular tachycardia (VT) and scar, using a lower voltage definition of the border-zone window.^1^ Ultimately, they found that the exit site of the VT and the border zone were in closer proximity to one another when the lower voltage definition was used.

While the study has significant limitations including the small number of patients enrolled, it remains important because it highlights two important points—that is, the usefulness of multielectrode mapping in VT ablation and the concept that the culprit abnormal area in VT is a relatively small core of the scar that can be potentially isolated as was described by Tzou et al. in 2015.^2^

Patients with structural heart disease referred for VT ablation often have comorbidities including congestive heart failure and kidney disease and, as a general finding, do not tolerate repeated induction and mapping of VT. Moreover, cardioversion/defibrillation shocks often lead to ventricular stunning and hemodynamic instability. Mechanical support can facilitate the ablation process but at the expense of increasing the complexity and cost of the procedure. As a result, the use of an ablation approach based on an accurate definition of the culprit scar and the ablation of the patient in sinus rhythm may reduce the safety and efficacy of the ablation procedure. While this concept is not new, combining it with multielectrode high-density mapping using a small interelectrode distance, a new definition of scar such as the one adopted in this study, and preprocedure scar imaging may further improve its value.

I hope that you enjoy reading the abovementioned article and that you find the contents of this issue of educational value.

Sincerely,


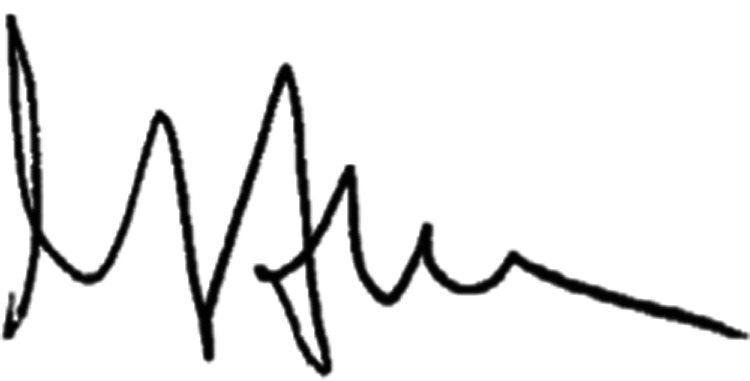


Moussa Mansour, MD, FHRS, FACC

Editor in Chief

The Journal of Innovations in Cardiac Rhythm Management

MMansour@InnovationsInCRM.com

Director, Atrial Fibrillation Program

Jeremy Ruskin and Dan Starks Endowed Chair in Cardiology

Massachusetts General Hospital

Boston, MA 02114
